# Commentary: Revisiting vocal perception in non-human animals: a review of vowel discrimination, speaker voice recognition, and speaker normalization

**DOI:** 10.3389/fpsyg.2015.00941

**Published:** 2015-07-07

**Authors:** Linda Polka, Ocke-Schwen Bohn, Daniel J. Weiss

**Affiliations:** ^1^School of Communication Sciences and Disorders, Centre for Research on Brain, Language, and Music, McGill UniversityMontreal, QC, Canada; ^2^Department of English and Center on Autobiographical Memory Research, Department of Psychology, Aarhus UniversityAarhus, Denmark; ^3^Department of Psychology and Program in Linguistics, The Pennsylvania State University, University ParkPennsylvania, PA, USA

**Keywords:** comparative research, vowel perception asymmetries, natural referent vowel framework, infants, non-human animals, task demands

Comparative research provides a unique window into our understanding of human vocal perception. We commend Kriengwatana, Escudero, and ten Cate (KEtC) for providing a much-needed review of this diverse literature. Their appraisal of three research areas highlights conceptual and empirical gaps, while also pointing to fruitful directions for future research.

This commentary addresses the literature on asymmetries in vowel perception. In their review of this topic KEtC focus on vowel contrasts that have revealed directional asymmetries in infants and non-human animals. We offer some clarification with respect to these stimulus issues and highlight another aspect of this research landscape—the role of task demands—that must also guide future comparative investigations.

## Vowel perception asymmetries—stimulus issues

The authors present a detailed overview of studies that reveal directional asymmetries in vowel discrimination in infants and several non-human species. To date infant perceptual asymmetries can be accounted for within the NRV framework (Polka and Bohn, 2011). Although directional asymmetries are evident for vowel pairs tested with cats, vervet monkeys, birds, and macaques, the overall pattern of the asymmetries observed in these species is inconsistent with the predictions of the NRV model^1^. As KEtC note, contrast-specific comparisons are limited because very few contrasts have been tested with both animals and infants. However, the richest cross-species data set pertains to the /ε-æ/ contrast. For this contrast, infants show the asymmetry predicted by NRV (easier in the /ε/ to /æ/ direction) whereas cats, birds, and vervets show an asymmetry in the opposite direction. Macaques performed at ceiling in both directions. These findings point to distinct vowel discrimination patterns in human infants and non-human animals. Surprisingly, KEtC dismiss these findings and question whether the /ε-æ/ asymmetry in infants is interpretable. They further suggest that we have not claimed that infant perception of /ε-æ/ supports the NRV framework; this is an incorrect representation of our work. In Polka and Bohn (1996), which subsequently led to the formulation of the NRV framework, we report and discuss the /ε-æ/ asymmetry and propose an account based on the location of these vowels in the vowel space (/æ/ is more extreme than /ε/). We further propose how this peripherality effect is acoustically grounded in Polka and Bohn (2011, p. 474, paragraphs 6, 7): “*The salience and stability of natural referent vowels is due to formant frequency convergence or focalization. …Focalization is graded and gives rise to salience differentials across the vowel space*.” To clarify, in all of the infant and animal experiments on the /ε-æ/ contrast to date, focalization differences are clearly observed; i.e., F1 and F2 are spectrally closer in the more peripheral vowel /æ/ compared to the less peripheral /ε/. Accordingly, there is no basis for viewing research on /ε-ae/ as irrelevant to a discussion of comparative differences in vowel perception asymmetries.

This issue aside, we concur with KEtC that the current literature is sparse and inadequate for drawing firm conclusions regarding species-specificity with respect to vowel perception asymmetries.

## Vowel perception asymmetries—task demands

In the current literature the tasks used to assess vowel discrimination asymmetries in infants and in other species are not comparable. These task discrepancies are not discussed by KEtC, yet they also severely limit the inferences that can be made. The animal studies cited by KEtC were conducted using psychophysical techniques designed to minimize cognitive resources making them ideal for comparing the peripheral sensory capacities of humans and non-human animals. In this work, a few subjects are extensively trained (with reinforcement) over many test sessions to discriminate a very small set of stimuli (one token per vowel), and memory demands are minimized by presenting the vowel stimuli with short inter-stimulus intervals (250–700 ms). This close temporal proximity allows the listener to access and compare acoustic details of the stimuli without encoding and retrieving information in a more enduring memory store. In contrast, the infant studies were conducted to understand how meaningful phonetic units are processed in more cognitively demanding tasks. Typically, a group of infants is tested using category-based discrimination tasks that involve stimulus variability (multiple tokens per vowel) and much less training, usually a single test session which may or may not involve reinforcement. Additionally, the temporal gaps between stimuli are longer (1000–1500 ms), placing higher demands on memory.

The animal research has focused on perception of just-noticeable differences while the infant research has focused on just-meaningful differences. This distinction is critical in the context of vowel perception asymmetries. In the NRV framework asymmetries reveal vowel perceptual biases that emerge when perceivers are accessing phonetic units, not simply detecting acoustic differences. Thus, phonetic biases are predicted to surface in tasks that mirror at least some of the demands of natural speech processing (high memory demands, stimulus uncertainty). The tasks implemented in the current animal literature clearly do not tap this level of processing. The psychophysical tasks implemented with animals would likely yield ceiling effects in humans which, interestingly, is the pattern found in macaques (Sinnott, 1989), a species with some ability to produce vowel-like sounds.

With respect to future research, we wholeheartedly agree with KEtC that testing human infants and non-human animals on the same vowel contrasts using comparable methods is required for drawing solid conclusions. More importantly, systematic manipulation of task demands is necessary to understand similarities and differences in the sensory, perceptual, and cognitive mechanisms across humans and non-human species. As highlighted by Weiss and Newport (2006) the perceptual and cognitive mechanisms that are fundamental to language acquisition cannot be adequately assessed with minimal stimulus variability/high training tasks. In the domain of vowel perception what is needed, as a minimum, are experiments that compare infants and other species in tasks that vary stimulus variability and memory load, and access to explicit training. Ideally, this would also involve comparing non-human primate species that vary in their capacity to produce vowel-like sounds.

Overall, when task differences are acknowledged, the current literature provides no compelling evidence that non-human animals show the kind of vowel perception biases that have been documented in humans. Despite the challenges, researchers are developing novel methods to assess perception in non-human animals across a wider range of processing demands. For example, several researchers have successfully measured spontaneous listening preferences in non-human primates (e.g., Watanabe and Nemoto, 1998; McDermott and Hauser, 2004). Understanding the evolution of language involves identifying which aspects of speech processing are shared with other animals and which are human-specific (Pinker and Jackendoff, 2005). To achieve this we must identify the dimensions of the speech signal that are accessed and also uncover the mechanisms that come into play when different species interact with spoken language.

## Conflict of interest statement

The authors declare that the research was conducted in the absence of any commercial or financial relationships that could be construed as a potential conflict of interest.

## Footnotes

^1^In KEtC's Figure 1, which plots asymmetries in discrimination of vervets from Sinnott (1989), the arrow going from /ʌ/to /ɔ/is in the wrong direction. The asymmetry described with respect to /u/-/℧/ is also reversed in the first paragraph on page three, but correctly displayed in Figure 1.
